# Double dermal sinuses: a case study

**DOI:** 10.1186/1752-1947-2-281

**Published:** 2008-08-26

**Authors:** Mostafa El Khashab, Farideh Nejat, Abolhasan Ertiaei

**Affiliations:** 1 Department of Neurosurgery, Hackensack University Medical Center, New Jersey, USA; 2 Department of Neurosurgery, Children's Hospital Medical Center, Medical Sciences/University of Tehran, Tehran, Iran

## Abstract

**Introduction:**

Dermal sinus tracts are rare congenital lesions located in the midline characterized by a cutaneous pit or dimple. They occur all along the midline neuroaxis, from the nasion and occipital area down to the lumbar and sacral regions, most frequently in the lumbar and lumbosacral region.

**Case presentation:**

Here we report a 5-year-old girl who presented with occasional headache. There were two dimples, one on the dorsal aspect of her head and another on her neck.

**Conclusion:**

Dermal sinuses are almost always singular and the co-existence of double dermal sinuses has not been reported previously.

## Introduction

Dermal sinus tracts are rare congenital lesions located in the midline characterized by a cutaneous pit or dimple. They are defined as developmental anomalies in which the end result can be abnormal communication between the dermis and intracranial structures. They incorporate a tract of cutaneous ectoderm from the dorsal midline skin that extends for a variable distance into the underlying mesenchymal tissue and in many instances penetrates the dura to end within the thecal sac adjacent to, or continuous with the neural tube [1]. Sinuses may be asymptomatic or present clinically with varying degrees of drainage from their cutaneous openings, recurrent bouts of septic or aseptic meningitis, or mass effect on the cerebrospinal fluid (CSF) pathways and consequent hydrocephalus [2].

These lesions are almost always solitary and co-existence of double dermal sinuses has not been reported previously. We report a girl with asymptomatic double dermal sinuses.

## Case presentation

**Figure 1:**
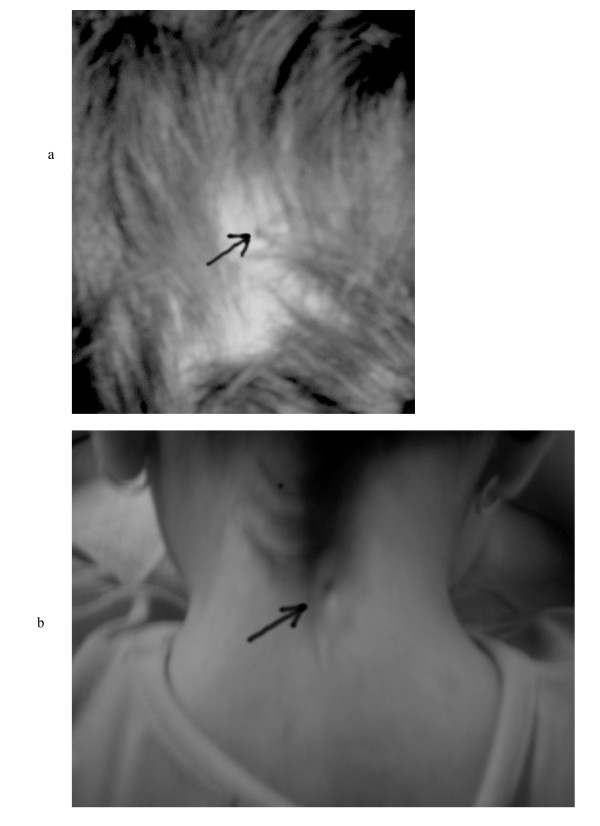
A: Photograph (posterior view) of the child's head showing a small opening in the midline occipital area *(arrow) *above the occipital protuberance and B: midcervical area.

**Figure 2:**
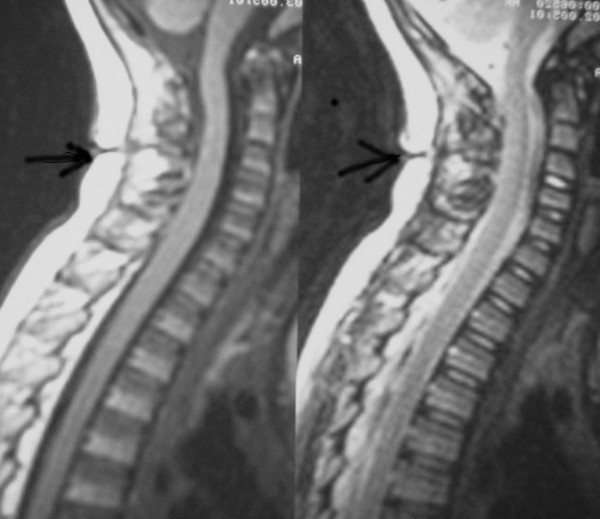
Sagittal T1-weighted MR image showing the opening of the dermal sinus at the level of C3–C4 and the extension of the tract outside the spinal canal.

This 5-year-old girl presented with occasional headache. She was the first child of nonconsanguineous parents without significant past medical history. On physical examination, the child was totally normal neurologically and generally. There were two dimples on the dorsal aspect of her head and neck. A fine dimple was noted at the midline occipital area above the inion, surrounded by a small smooth hairless area, harboring a few thick black hairs at the ostium without any discharge (Fig. 1a). The other dimple was at the midcervical area with a large mouth and hemangiomatous skin discoloration around the dimple (Fig. 1b). Brain magnetic resonance imaging (MRI) was performed, which was normal without bone defect and intracranial sinus or tract. Cervical MRI showed the sinus at the level of the C3–C4 vertebra with a tract ending before the spinal canal (Fig. 2). She had not experienced any previous infection and there was no intradural extension for both lesions, therefore further investigation or procedure was not done.

## Discussion

Congenital dermal sinus is a rare entity (1/2500), and consists of a tract lined by stratified squamous epithelium [3,4]. It is often detected at birth, and usually by the end of the first year. Typically, the pediatrician notes a midline dimple or cutaneous defect, containing one or more hairs [5].

The most widely accepted theory regarding the embryogenesis of dermal sinus tracts and related anomalies proposed that they arise through faulty separation of the neuroectoderm from the overlying cutaneous ectoderm at the time of dysjunction between the third and eighth week of gestation [1,5,6].

They have been reported all along the midline neuroaxis, from the nasion and occipital area down to the lumbar and sacral regions [3], most frequently in the lumbar and lumbosacral region (75%) and only 1% of all tracts along the spine are cervical [6,7].

Cranial sinuses are less frequent than their counterparts in the spinal region in which 85% are located near the external protuberance of the occipital bone, 11% at the nasion and 5% at the posterior parietal area [2]. The sinus tract may end in subcutaneous tissue or extend any distance inward to its ultimate embryological terminus, which is the conus medullaris for lesions in the lumbosacral region or the central canal of the spinal cord for tracts at the thoracic or cervical level [3].

Approximately one-half of the tracts terminate in a dermoid (83%) or epidermoid (13%) cyst or a teratoma (4%). The slow growth rate of these tumors often masks their presentation for years, although there are some patients with acute neurological deterioration [3,8].

A wide spectrum of clinical manifestations can occur ranging from asymptomatic dermal sinus to serious complications. There is no apparent timeframe for an asymptomatic lesion to later become symptomatic [9]. This process is believed to be benign until an episode of meningitis [5]. Meningitis resulting from dermal sinus tracts may occur at any age and is seen in infants and elderly patients. Patients may present with concomitant infections of the dermal sinus tract and underlying inclusion cysts [8].

MRI has become the reference study technique because of its ability to accurately depict the extent of the sinus tract and associated lesions [8].

Therapy is almost always surgical. The goal is obliteration of the tract with elimination of the communication between the skin and the neural structures. The earlier the lesion is detected and corrected, the less likely any long-term morbidity [5].

The process of dysjunction occurs after closure of the neural tube at a time between the third and eighth week of gestation, whereas it develops during the third to fifth week of intrauterine life in the cranium. At the same time, the cutaneous portion of the neuroectoderm separates and fuses in the midline to form the overlying integument. Disorders of this process may lead to midline dermal anomalies such as dermal sinus tracts and inclusion cysts [5,6,8].

## Conclusion

We described a rare case of double dermal sinus. To our knowledge, there is no report of double or multiple dermal sinuses in the literature. Regarding the similar range of gestational age for dysjunction in the spinal and cranial neural tube, the occurrence of double dermal sinuses in one person, of which one is cranial and the other cervical, suggests that there is an underlying cause which affects separation of the neuroectoderm at an early gestational age during the third to eighth week of gestation.

## Competing interests

The authors declare that they have no competing interests.

## Authors' contributions

All authors have contributed to the study and manuscript preparation.

## Consent

Written informed consent was obtained from the patient's next of kin for publication of this case report and any accompanying images. A copy of the written consent is available for review by the Editor-in-Chief of this journal.
